# Corrigendum: Extracorporeal Shock Wave Therapy Enhances the *In Vitro* Metabolic Activity and Differentiation of Equine Umbilical Cord Blood Mesenchymal Stromal Cells

**DOI:** 10.3389/fvets.2022.840356

**Published:** 2022-01-28

**Authors:** Ramés Salcedo-Jiménez, Judith B. Koenig, Olivia J. Lee, Thomas W. G. Gibson, Pavneesh Madan, Thomas G. Koch

**Affiliations:** ^1^Department of Clinical Studies, University of Guelph, Guelph, ON, Canada; ^2^Department of Biomedical Sciences, University of Guelph, Guelph, ON, Canada

**Keywords:** shock wave, mesenchymal stromal cells, equine stem cells, umbilical cord, immunomodulatory

In the original article, there was a mistake in Figure 6 as the cartilage histology images (ESWT – and ESWT +) were the same image. The corrected Figure 6 appears below.

**Figure 6:**
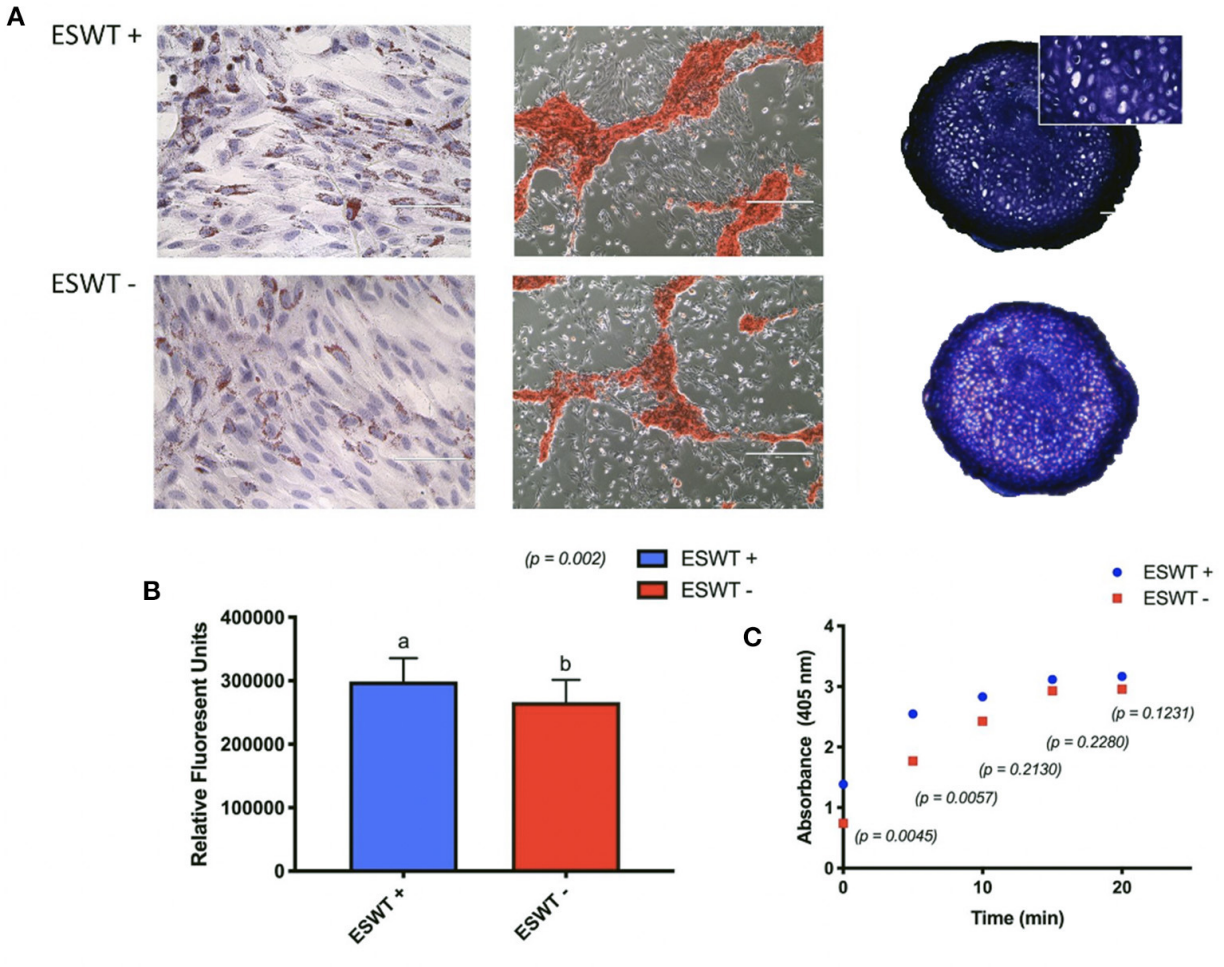
Trilineage differentiation. **(A)** Positive trilineage potency for adipogenesis (Oil Red O), osteogenesis (Alizarin Red S), and chondrogenesis (Toluidine blue) for treated (ESWT+) and untreated (ESWT–) equine CB-MSC. **(B)** Quantification of intracellular lipid droplets by relative fluorescent units for treated (ESWT+) and untreated (ESWT–) equine CB-MSC. Results are shown as mean and CI 95% (*p* = 0.0002). Values with different letters are statistically different (*p* = 0.002). **(C)** Alkaline phosphatase activity measured in relative fluorescent units in treated (ESWT+) and untreated (ESWT–) equine CB-MSC. Differences were found at time 0 (*p* = 0.0045) and 5 mins (*p* = 0.0057).

The authors apologize for this error and state that this does not change the scientific conclusions of the article in any way. The original article has been updated.

## Publisher's Note

All claims expressed in this article are solely those of the authors and do not necessarily represent those of their affiliated organizations, or those of the publisher, the editors and the reviewers. Any product that may be evaluated in this article, or claim that may be made by its manufacturer, is not guaranteed or endorsed by the publisher.

